# Attenuation of Drought Stress in *Brassica* Seedlings with Exogenous Application of Ca^2+^ and H_2_O_2_

**DOI:** 10.3390/plants6020020

**Published:** 2017-05-13

**Authors:** Akram Khan, Yasir Anwar, Md. Mahadi Hasan, Aqib Iqbal, Muhammad Ali, Hesham F. Alharby, Khalid Rehman Hakeem, Mirza Hasanuzzaman

**Affiliations:** 1 Institute of Biotechnology and Genetic Engineering, University of Agriculture, Peshawar 25120, Pakistan; akramehe@gmail.com (A.K.); spark.jk@gmail.com (A.I.); 2 Department of Biotechnology, Bacha Khan University Charsadda, Charsadda 24420, Pakistan; drali@bkuc.edu.pk; 3 Department of Biological Sciences, Faculty of Science, King Abdulaziz University, Jeddah 21589, Saudi Arabia; yasirpcsir2006@gmail.com (Y.A.); mahadicubono@yahoo.com (M.M.H.); halharby@kau.edu.sa (H.F.A.); 4 Department of Agronomy, Faculty of Agriculture, Sher-e-Bangla Agricultural University, Sher-e-Bangla Nagar, Dhaka 1207, Bangladesh; mhzsauag@yahoo.com

**Keywords:** drought stress, relative water content, electrolyte leakage, proline, SDS-PAGE

## Abstract

Drought is one of the most common abiotic stresses, affecting the growth and productivity of crop plants globally, particularly in arid and semi-arid regions. Different strategies are used to mitigate the impact of drought among crop plants. Exogenous application of different substances are known to decrease the effects of various abiotic stresses, including drought stress. The aim of this study was to evaluate the effect of Ca^2+^ and H_2_O_2_ in developing drought stress tolerance in *Brassica napus* “Bulbul-98” seedlings. *Brassica napus* “Bulbul-98” seedlings were exposed to 5, 10 and 15 mM Ca^2+^ and 2, 5 and 10 μM H_2_O_2_ concentrations twice at an interval of two days for up to 20 days after germination. Drought stress decreased relative water content (RWC), chlorophyll content and increased proline, H_2_O_2_, soluble protein and electrolyte leakage in *Brassica* seedlings. Exogenous Ca^2+^ (5, 10,15 mM) and H_2_O_2_ (2, 5, 10 μM) supplementations, during drought stress induction, showed a significant increase in RWC by 5.4%, 18.06%, 26.2% and 6.87%, 13.9%, 18.3% respectively. Similarly, with the exogenous application of Ca^2+^ (5, 10, 15 mM) and H_2_O_2_ (2, 5, 10 μM), chlorophyll content was increased by 15.03%, 22.2%, and 28.4%, and 9.6%, 23.3%, and 27.5% respectively. It was confirmed that the seedlings under drought stress that were supplemented with Ca^2+^ and H_2_O_2_ recovered from water content reduction and chlorosis, and were able to grow normally.

## 1. Introduction

A number of abiotic stresses like drought, temperature, and salinity, usually reduce crop yield [1]. It has been approximated that crops reachonly 25% of their likely yield, because of the damaging effects of environmental stresses [2]. These stresses can take place at any phase of plant growth, thus illustrating the dynamic nature of crop plants and their yield. Drought is one of the main abiotic stresses, and significantly affects yield and growth of plants, and plays a vital role in their geographical division [3,4,5]. According to the Food and Agriculture Organization (FAO), 45% of the agricultural land on earth is exposed to drought stress [6]. Water deficiency induces a set of physiological and biochemical reactions in plants and is one of the most composite unfavorable conditions, since it not only depends on the severity and period of the stress occurrence, but also on the plant developmental period and its morphology [7,8]. As an adaptive and protection mechanism, plant hormonal and signaling networks are involved in various ways to manage stress under various abiotic stress conditions [9]. Even though an assortment of genotypes with improved yield in drought conditions has been a vital feature of crop reproduction, the biological basis for drought tolerance is still poorly understood. High photosynthesis rate maintenance [10], osmotic modification to decrease water loss [11], high instantaneous water effectiveness maintenance (defined as the ratio of transpiration to leaf photosynthesis) [12], waxy coatings on the plant exterior, and deeper root morphologies, are some of the traits found in drought tolerant genotypes. The inhibition of development leading to the production of a range of modifications in plant physiological, biochemical and molecular features is generally caused by drought stress [3,4,5].

A general occurrence in plants subjected to various abiotic and biotic stresses, is the production of reactive oxygen species (ROS). By the commencement of an antioxidant defense system consisting of enzymatic and non-enzymatic components, the cells usually retain a stable-state ROS level [13]. ROS are greatly reactiveto DNA, membrane lipids, and protein, and they are key causative factors for stress-induced cellular injures. High antioxidant ability or high levels of antioxidants can avoid cell death and is associated with stress tolerance [14,15]. Several studies have shown that H_2_O_2_, one of the mobile forms of ROS, is a major signal molecule, mediating a seriesof reactions [16]. Exogenous Ca^2+^ can improve plant stress resistance, guard the structure of cellular plasma membranes, slow down the synthesis of activating oxides, control the metabolism of plant hormones, and sustain normal photosynthesis [17,18,19]. Besides this, cellular Ca^2+^ also transmits drought signals, therefore modifying physiological reactions introduced by drought stress [20,21]. Related results of improved stress tolerance have been observed subsequent to pre-treatment with H_2_O_2_ [22,23].

Among the oilseeds crops, *Brassica* is one of the most important crops, due to its edible oil production [24]. *Brassica* has been developed in high rainfall areas, and does not grow well in low rainfall areas [25]. Reduction of the yield of *Brassica* due to drought stress have been reported by many authors [26,27]. However, the influence of various exogenous elements in the reduction of drought stress is still in its infancy.

Hence, the current study was undertaken to find out the consequences of the exogenous application of Ca^2+^ and H_2_O_2_ pre-treatment on the drought stress tolerance of *Brassica napus* “Bulbul-98” at early growth stages. This study has also elucidated the physiological and biochemical changes under drought stress conditions associated with the pre-treatment of these chemicals and determination of differentially expressed proteins with these pre-treatments under normal (irrigated) and drought stress conditions.

## 2. Results

### 2.1. Rate of Water Loss (RWL)

**Figure 1:**
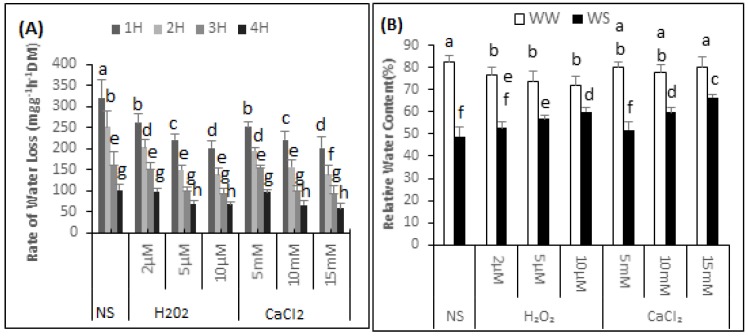
H_2_O_2_ and CaCl_2_ pre-treatment effect on the of the rate of water loss (**A**) over time (h, 1H, 2H, 3H and 4H); Relative water content (**B**) of *Brassica napus* “Bulbul-98” seedlings under irrigated (WW) and drought stress (WS) conditions (**B**). In accordance with Least Significant Difference (LSD) test, the bars with at least one common alphabet are not significantly different at *p* ≤ 0.05.

The highest rate of water loss was 320.45 mg·g^−1^h^−1^ (DM) found in the non-supplemented seedlings (Figure 1A). During the 1 h measuring period, the rate of water loss of the seedlings supplemented with 2, 5 and 10 μM H_2_O_2_ decreased by 18.5%, 31.1% and 37.18% respectively. The water loss rate from leaf discs of the seedlings sprayed with 5, 10 or 15 mM CaCl_2_ was 252.45, 221.4 and 202.3 mg·g^−1^h^−1^ (DM) respectively.

### 2.2. Relative Water Content (RWC)

The data showed that the RWC of the non-supplemented (NS) seedlings was 82.39% ± 3.13% under irrigated conditions and 49.02% ± 4.20% after exposure to drought stress. Thus there was 40.5% decrease in the RWC of the non-supplemented seedlings under drought stress conditions. It can be further observed from the data that H_2_O_2_ application significantly reduced RWC under irrigated conditions (Figure 1B). The RWC of the seedlings supplemented with 2, 5 and 10 μM H_2_O_2_ was 76.49% ± 3.53%, 73.99% ± 4.27% and 72.19% ± 3.67%, respectively. Thus, compared with seedling that were non-supplemented in irrigated conditions, the RWC of the seedlings supplemented with 2, 5 and 10 μM H_2_O_2_ decreased by 7%, 10% and 12%, respectively. In contrast, when exposed to drought stress situations, there was a gradual increase in the RWC of the seedlings supplemented with H_2_O_2_, compared with non-supplemented seedlings. The RWC of the seedlings exposed to drought stress and supplemented with 2, 5, and 10 μM H_2_O_2_, was 52.64% ± 2.79%, 56.95% ± 1.38% and 60.01% ± 1.68%. Thus, compared with non-supplemented seedlings, there was an increase of 6.87%, 13.9% and 18.3% in the RWC of seedlings under drought stress supplemented with 2, 5, and 10 μM H_2_O_2_, respectively. There was no major effect on the RWC of seedlings under irrigated conditions after supplementation with CaCl_2_. The “Bulbul-98” seedlings maintained a RWC of 80.06% ± 2.67%, 78.04% ± 3.29% and 80.25% ± 4.68%, respectively under irrigated conditions after supplementation with 5, 10 and 15 mM of CaCl_2_. Spraying the seedlings with 5, 10 and 15 mM CaCl_2_ prior to exposure to drought gradually increased the RWC to 51.85% ± 3.79%, 59.83% ± 1.95% and 66.37% ± 1.63%.

### 2.3. Chlorophyll Content

**Figure 2:**
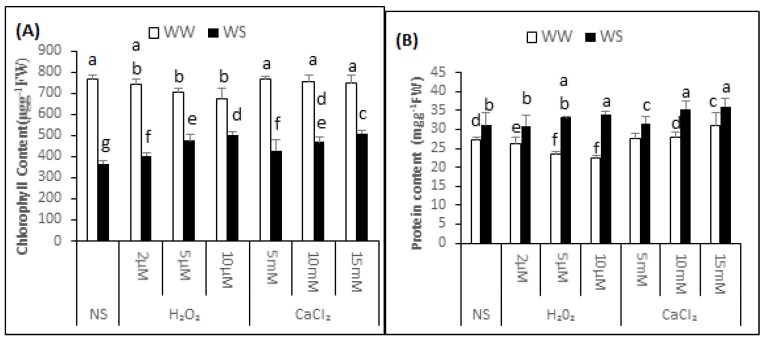
H_2_O_2_ and CaCl_2_ supplementation effect on the content of chlorophyll (**A**) and protein content (**B**) of the *Brassica napus* “Bulbul-98” seedlings under irrigated (WW) and drought stress (WS) conditions. In accordance to Least Significant Difference (LSD) test, the bars with at least one common alphabet are not significantly different at *p* ≤ 0.05.

Mean chlorophyll content of the non-supplemented (NS) seedlings was 768.20 ± 19.58 μg·g^−1^ fresh weight (FW) under irrigated conditions and 364.96 ± 14.53 μg·g^−1^ FW after exposure to drought stress. Thus, the exposure to drought stress decreased the chlorophyll content by 53%, signifying enhanced modification to chloroplast (Figure 2A). The content of chlorophyll was 743.09 ± 25.51, 704.48 ± 15.54 and 674.03 ± 48.82 μg·g^−1^ FW in the seedlings treated with 2, 5 and 10μM H_2_O_2_. Thus, H_2_O_2_ application upon irrigated situations has enhanced alteration to chlorophyll in the seedlings. After 2, 5 and 10 μM H_2_O_2_ application, chlorophyll content was 403.01 ± 15.56, 475.90 ± 29.21 and 503.67 ± 13.98 μg·g^−1^ FW under drought stress conditions. This data indicated that there was 10%, 25% and 29% less modification to chlorophyll respectively, in the supplemented seedlings compared with non-supplemented seedlings under similar conditions. Before applying the drought, the chlorophyll content was 429.53 ± 49.10, 469.13 ± 24.27 and 519.94 ± 10.47 μg·g^−1^ FW under 5, 10 and 15 mM CaCl_2_ treatment. Thus the seedlings supplemented with CaCl_2_ before drought imposition incurred 13%, 24% and 33% less modification to chlorophyll compared with non-supplemented seedlings.

### 2.4. Soluble Protein Content

In the non-supplemented seedlings, the protein content was 26.42 ± 1.40 and 31.30 ± 2.29 mg·g^−1^ FW in irrigated and drought stress conditions, respectively (Figure 2B). The protein content was 26.17 ± 1.80, 25.66 ± 1.36 and 25.33 ± 0.38 mg·g^−1^ FW in irrigated and 30.70 ± 2.09, 32.82 ± 0.45 and 32.34 ± 2.78 mg·g^−1^ FW in drought stress conditions after pre-treatment of the seedlings with 2, 5 and 10 μM H_2_O_2_, respectively. Thus, compared with non-supplemented seedlings, there was a 0.94%, 2.90% and 4.25% decrease in the soluble protein content under irrigated conditions and an initial 1.92% decrease, then a 4.95% and 3.17% increase in protein content under drought stress conditions after 2, 5 and 10 μM H_2_O_2_ pre-treatment, respectively. Though statistically non-significant, pre-treatment of the “Bulbul-98” seedlings with CaCl_2_ resulted in induction of soluble protein accumulation under both irrigated and drought stress conditions. The protein content was 27.55 ± 1.38, 27.84 ± 1.31 and 30.45 ± 2.50 mg·g^−1^ FW in irrigated and 33.06 ± 2.79, 34.36 ± 0.54 and 35.97 ± 1.50 mg·g^−1^ FW under drought stress conditions after pre-treatment of the seedlings with 5, 10 and 15 mM CaCl_2_, respectively. Thus, compared with the non-supplemented seedlings under respective conditions, pre-treatment with 5, 10 and 15 mM CaCl_2_ increased the protein content by 4.28%, 5.15% and 14.48% under irrigation, and 5.62%, 9.26% and 13.59% underdrought stress conditions, respectively.

### 2.5. Electrolyte Leakage

**Figure 3:**
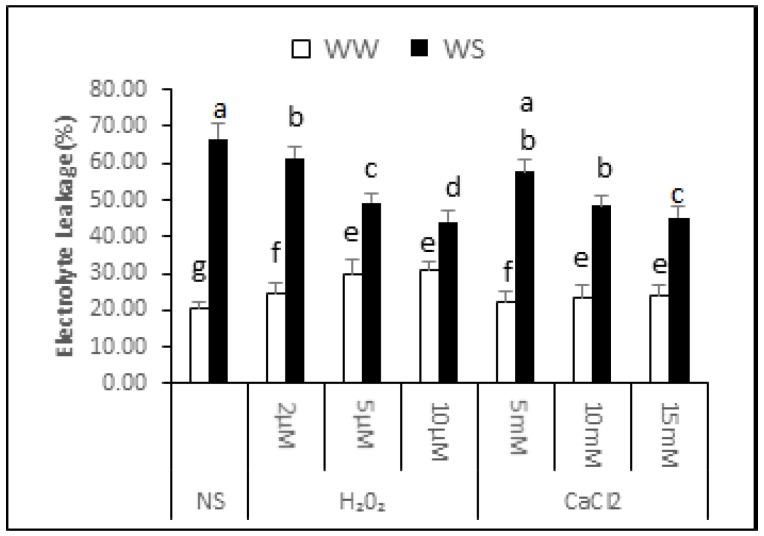
Effect of H_2_O_2_ and CaCl_2_ supplementation on percent electrolyte leakage (%) from *Brassica napus* “Bulbul-98” seedlings under irrigated (WW) and drought stress (WS) conditions. In accordance with the Least Significant Difference (LSD) test, the bars with at least one common alphabet are not significantly different at *p* ≤ 0.05.

The data showed that electrolyte leakage of the non-supplemented (NS) seedlings was 20.74% ± 1.65% under irrigated conditions and 66.60% ± 4.35% after exposure to drought stress. Thus, exposure to drought stress increased the electrolyte leakage by 2.21-fold, signifying an increase in membrane damage (Figure 3). In irrigated conditions, increasing H_2_O_2_ concentration supplementation gradually increased electrolyte leakage. Electrolyte leakage was 24.64% ± 2.90%, 29.64% ± 4.15% and 30.94% ± 2.16% in seedlings treated with 2, 5 and 10 μM H_2_O_2_, respectively. Thus, application of H_2_O_2_ under irrigated situations enhances damage to cellular membranes, resulting in increased water loss and lower relative seedling water content. CaCl_2_ supplementation, on the other hand, was not significantly affected for electrolyte leakage in irrigated conditions. It can be noted from the data that application of H_2_O_2_ or CaCl_2_ upon drought stress conditions partly decreased electrolyte leakage, signifying lesser damage to cellular membranes. After 2, 5 and 10 μM application of H_2_O_2_, the electrolyte leakage was 61.37% ± 3.06%, 49.27% ± 2.59% and 44.18% ± 2.70% under drought stress conditions respectively. This indicated that there was 8%, 26% and 34% less electrolyte leakage from the supplemented seedlings compared with non-supplemented seedlings under similar conditions. Similarly, the electrolyte leakage in seedlings applied 5, 10 and 15 mM CaCl_2_ before drought was 57.54% ± 3.28%, 48.33% ± 2.91% and 45.26% ± 3.04%, respectively. The seedlings supplemented with CaCl_2_ before drought treatment in curred 14%, 27% and 32% less membrane damage compared with non-supplemented seedlings.

### 2.6. Proline Content

**Figure 4:**
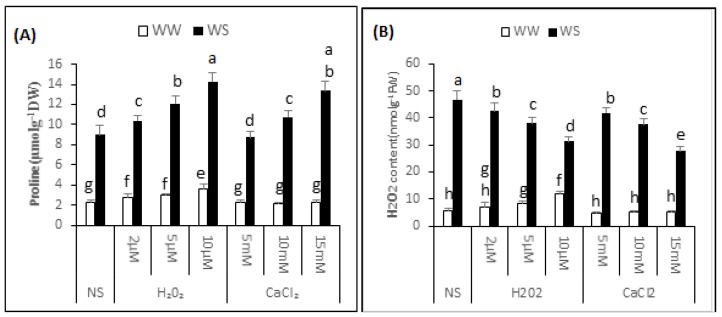
H_2_O_2_ and CaCl_2_ supplementation effect on the proline (**A**) and H_2_O_2_ content (**B**) of the *Brassica napus* “Bulbul-98” seedlings under irrigated (WW) and drought stress (WS) conditions. In accordance to Least Significant Difference (LSD) test, the bars with at least one common alphabet are not significantly different at *p* ≤ 0.05.

Variance analysis exhibited a significant difference (*p* < 0.05) in the proline content of “Bulbul-98” seedlings following H_2_O_2_ or CaCl_2_ supplementation under both irrigated and drought stress conditions (Figure 4A). In the non-supplemented seedlings, the proline content increased to 8.99 ± 0.89 from 2.27 ± 0.28 μmol·g^−1^ DW. In irrigated conditions, a major increase in proline content was noted with increasing quantity of supplemented H_2_O_2_. Thus, the proline content was 2.79 ± 0.37, 2.98 ± 0.18 and 3.55 ± 0.55 μmol·g^−1^ DW in the seedlings regularly irrigated and with 2, 5 and 10 μM H_2_O_2_ respectively. CaCl_2_, in contrast, did not significantly affect the proline content in irrigated conditions. A significant induction of proline was noted with H_2_O_2_ or CaCl_2_ application under drought stress conditions. The application of 2, 5 and 10 μM H_2_O_2_ improved the proline content to 10.35 ± 0.58, 12.09 ± 0.79 and 14.31 ± 0.88 μmol·g^−1^ DW respectively, and 5, 10 and 15 mM CaCl_2_ application before drought increased the proline content to 8.75 ± 0.62, 10.74 ± 0.58 and 13.38 ± 0.90 μmol·g^−1^ DW, respectively. Thus, application of 2, 5 and 10 μM H_2_O_2_ improved the proline content of seedlings by 15%, 34% and 59%, and 10 and 15 mM CaCl_2_ increased the proline content by 20% and 49% respectively, over non-supplemented seedlings, under similar conditions.

### 2.7. H_2_O_2_ Content

The analysis of variance exhibited a significant difference (*p* ≤ 0.05) in endogenous H_2_O_2_ content of “Bulbul-98” seedlings after H_2_O_2_ or CaCl_2_ supplementation in both irrigated and drought stress conditions (Figure 4B). In the non-supplemented seedlings, drought stress conditions improved the content of H_2_O_2_ to 46.46 ± 3.68 from 6.29 ± 0.55 nmol·g^−1^ FW. In irrigated conditions, a major increase in endogenous content of H_2_O_2_ was noted with an increasing amount of supplemented H_2_O_2_, and 8.35 ± 0.84, 9.82 ± 0.76 and 11.44 ± 0.52 nmol·g^−1^ FW H_2_O_2_ was recorded in the seedlings that were regularly irrigated and treated with 2, 5 and 10 μM H_2_O_2_. Seedlings pre-treated with CaCl_2_, in contrast, were not considerably affected in endogenous H_2_O_2_ content under irrigated conditions. A significant interactive effect of pre-treatment of seedlings with H_2_O_2_ or CaCl_2_ application and drought stress was noted with regards to the endogenous content of H_2_O_2_. Compared with non-supplemented seedlings under drought stress conditions, a decrease in content of endogenous H_2_O_2_ was observed with increased amounts of H_2_O_2_ or CaCl_2_. The content of endogenous H_2_O_2_ under drought stress was 41.77 ± 3.68, 35.08 ± 1.46 and 28.68 ± 1.24 nmol·g^−1^ FW, respectively after pre-treatment of seedlings with 2, 5 and 10 μM H_2_O_2_ before exposure to drought. Similarly, pre-treatment of the seedlings with 5, 10 and 15 mM CaCl_2_ application before drought decreased the content of endogenous H_2_O_2_ to 40.66 ± 0.37, 37.26 ± 2.22 and 27.04 ± 3.97 nmol·g^−1^ FW, respectively. Thus, application of 2, 5 and 10 μM H_2_O_2_ decreased the content of endogenous H_2_O_2_ in the seedlings by 10%, 24% and 38%respectively, and 5, 10 and 15 mM CaCl_2_ decreased the content of endogenous H_2_O_2_ by 12%, 20% and 42% respectively, over the non-supplemented seedlings under similar conditions.

### 2.8. SDS-PAGE Analysis

**Figure 5:**
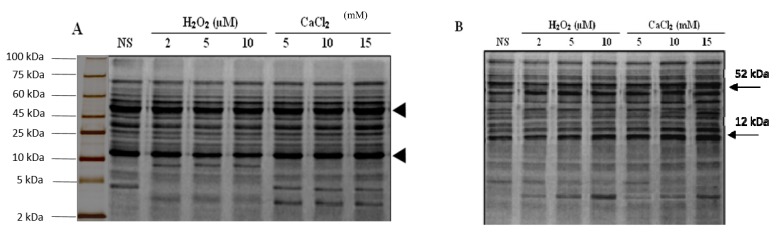
SDS-PAGE electrophoregram of total soluble proteins of “Bulbul-98” seedlings under irrigated (**A**) and drought stress (**B**) conditions after H_2_O_2_ and CaCl_2_ pre-treatment. Upper and lower arrowheads in each electrophoregram shows the large and small subunits of Rubisco. NS, non-supplemented.

Total soluble proteins from the non-supplemented and the seedlings pre-treated with different concentrations of H_2_O_2_ and CaCl_2_ under irrigated and drought stress conditions were separated through a one dimensional 15% sodium dodecyl sulfate polyacrylamide gel electrophoresis (SDS-PAGE) (Figure 5). It was observed from the intensity of the bands, that approximately equal amounts of protein were loaded in each well. The protein bands obtained through the SDS-PAGE gels from seedlings exposed to different treatments were quantified through computer software BandLeader^®^, and this was also visually confirmed. The data clearly indicated that both qualitative and quantitative changes occurred in the seedlings as a result of water availability and different treatments. A total of 20 protein bands were identified by the Band Leader^®^ software in the non-supplemented seedlings under irrigated conditions. Quantitative changes were noted in the SDS-PAGE banding pattern of the seedlings regularly irrigated after pre-treatment with H_2_O_2_ or CaCl_2_ did not affect the protein content, as predicted from the band intensities; however, an increase in the protein concentration was noted after 10 and 15 mM CaCl_2_ application. Rubisco large and small sub-units (RbcL and RbcS, respectively) were the most abundant proteins in the gel under all conditions (Bands No. 5 and 12). Band 5 and Band 12 had intensities of approximately 150 pixels in the non-supplemented seedlings, and those pre-treated with 2, 5 and 10 μM H_2_O_2_ and 5 mM CaCl_2_under irrigated conditions. Related to the increase in protein content, Band 5 and 12 intensities have increased to 220 pixels after pre-treating the seedlings with 10 and 15 mM CaCl_2_, respectively. Similarly, there was a slight decrease in the intensity of band 7 after H_2_O_2_ but this increased after CaCl_2_ pre-treatment under irrigated conditions. The intensity of this band was 135 pixels in the non-supplemented seedlings, and decreased to 124 pixels after H_2_O_2_ pre-treatment, and increased to 190 and 210 pixels after 10 and 15 mM CaCl_2_ pre-treatment. The intensity of band 18 also increased after both H_2_O_2_ and CaCl_2_ pre-treatments. The disappearance of band 17 after H_2_O_2_ pre-treatment was the only qualitative difference in the SDS-PAGE. When exposed to drought stress, a total of 23 bands were observed in the non-supplemented seedlings (Figure 5B). Among these, 19 bands were those also expressed in the non-supplemented seedlings under irrigated conditions. Band 17 in the irrigated conditions was not expressed under the drought stress conditions. However, an increase was observed in the intensity of most of the bands. Band 12 had intensities of approximately 130 pixels in the non-supplemented seedlings, and those pre-treated with 2, 5 and 10 μM H_2_O_2_, and 5 mM CaCl_2_ under drought stress conditions.

Band 5 and 12 intensities increased to 190 pixels after pre-treating the seedlings with 10 and 15 mM CaCl_2_, respectively. However, there was a slight increase in the intensity of several bands after CaCl_2_ pre-treatment under drought stress conditions. The intensity of these band were between 100–110 pixels in the non-supplemented seedlings, as well as those pre-treated with 2, 5 and 10 μM H_2_O_2_ and 5 mM CaCl_2_ which increase to 170 pixels after 10 mM CaCl_2_, and gradually increased to 190 pixels after 15 mM CaCl_2_ pre-treatment under drought stress conditions.

## 3. Discussion

Evidence showed that H_2_O_2_ influences the activation or inhibition of various cellular processes in a dose-dependent manner. H_2_O_2_atlow concentration enhances plant tolerance to a range of abiotic and biotic stresses [28]. Similarly, Ca^2+^ acts as a secondary messenger to couple a large variety of extra-cellular stimuli with intracellular responses in plant cells. It also has a stabilizing effect on cell wall and membranes, and improves the drought tolerance of plant cells [19,29]. Though CaCl_2_ is lethal to plants in higher concentrations, in low quantities it may enhance stress tolerance by the provision of Ca^2+^ for cell stabilization and signaling, thus inducing the production of different stress peptides. Furthermore, Ca^2+^ and Cl^−^ are also essential cofactors for photosynthetic water oxidation. This experiment was designed to study whether pre-treatment with H_2_O_2_ or CaCl_2_activates plant signaling mechanisms and acclimatizes the seedlings under drought stress conditions. The initial reaction of plants to a diminishing water supply is reduction of water loss, which is attained by either the stomata closing, or reducing the potential of water by accumulation of different solutes. In this experiment, the decrease in water loss rate from excised leaves was first measured to ascertain the beneficial effect of H_2_O_2_ and CaCl_2_ pre-treatment before drought imposition. RWL has been suggested as a screening technique to identify genotypes under drought stress [30]. The data suggest that H_2_O_2_ and CaCl_2_ pre-treatments both resulted in significantly reduced water loss from leaf disks in a dose dependent manner (Figure 1A). The excised leaves from the non-supplemented “Bulbul-98” had a significantly higher rate of water loss compared with water loss from leaves of H_2_O_2_ and CaCl_2_-treated plants. A similar difference in the rate of water loss from untreated plants and those exposed to periodic drought has been observed in tobacco [31]. Different factors including the opening of stomata, accumulation of compatible proteins and solutes, protection of cellular membranes from lipid peroxidation, and deposition of cuticular waxes could affect the water loss rate from a plant. The physiological adaptation of “Bulbul-98” pre-treated with H_2_O_2_ or CaCl_2_ before drought imposition was further probed by determining the relative water content of the leaves from seedling under each treatment. The relative water content changes also reflected the ameliorative effect of H_2_O_2_ and CaCl_2_ pre-treatment before drought stress (Figure 1B). It could also be noted that H_2_O_2_ application under irrigated conditions resulted in a decrease in RWC, but there was no adverse effect of CaCl_2_ pre-treatment under similar conditions. Furthermore, the ameliorative effect of CaCl_2_ treatment on the RWC was more pronounced under drought stress conditions, compared with pre-treatment of H_2_O_2_. Similar to our results, the improvement of water relations after H_2_O_2_ pre-treatment in maize [32] and *Cistus albidus* [33] under drought and soybean [34] under salinity stress has been reported. Furthermore, improved water relations have been reported after pre-treatment with CaCl_2_ under salinity and flooding stress in wheat, rice, and barley. It is known that under drought stress conditions, a positive turgor pressure is maintained by stomatal closure [35] or osmotic adjustment, through the accumulation of compatible solutes [36]. The results of RWL and RWC in drought- stressed seedlings after H_2_O_2_ or CaCl_2_ pre-treatment indicated an improvement in water relations. However, if this improvement is only due to stomatal closure, it will typically induce the limitation of gas exchange and alter the rate of photosynthesis and metabolism [37]. It is known that H_2_O_2_ triggers proline accumulation in maize seedlings, a compatible solute [38]. Similarly, elevated content of proline and glycine betaine, improved the water status and resulted in minimum damage to cellular membranes, and Ca^2+^ in the medium appeared to reduce damaging effect of stress [39]. To properly understand the physiological mechanism of tolerance after H_2_O_2_ and CaCl_2_ pre-treatment, the concentration of osmoprotectant proline was determined under control and drought stress conditions. A minor increase in proline content was noted in seedlings pre-treated with H_2_O_2_ in the irrigated conditions, showing a minor alteration of cellular metabolism and induction of stress (Figure 4A). However, the data showed a significant increase in proline content upon conditions of drought stress. Furthermore, the application of H_2_O_2_ or CaCl_2_ upon conditions of drought stress strongly induced proline production. This increase in the proline content could be due to the induction of a proline-producing enzyme and the inhibition of the catabolic enzyme proline oxidase. The proline content increase under drought helps with osmotic adjustment. Though the data at the last of drought stage indicated a lower rate of water loss with pre-treatments, it appears that the pre-treated seedlings maintained a steady state of transpiration compared with non-supplemented seedlings. The non-supplemented seedlings maintained a higher transpiration, resulting in depletion of water and enhanced damage to cells. Thus, this data provided a further proof of the hypothesis that H_2_O_2_ or CaCl_2_applications induces proline production under drought stress conditions. Stressful conditions induce complex and highly regulated ROS accumulation through plasma membrane-bound NADPH oxidase and NADPH peroxidase of cell walls. This ROS accumulation, especially H_2_O_2_, stimulates or down-regulate differently located enzymes, some of which are involved in H_2_O_2_ generation & degradation [40]. Furthermore, the increased production of the hydroxyl radical (·OH), induces lipid peroxidation, resulting in damage to cellular membranes [41]. The damage to the membranes results in the uncontrolled loss of water and nutrients, and entry of extracellular hydrolases, thus adversely affecting cellular metabolism [42]. The physiological response of the “Bulbul-98”; seedlings to drought imposition after each pre-treatment was further elucidated with the determination of damage to cellular membranes and concentration of endogenous H_2_O_2_. The data showed an enhancement in electrolyte leakage from the membranes, and endogenous H_2_O_2_ concentration after exposure of “Bulbul-98” seedlings to drought stress conditions (Figure 3). The pre-treatment of seedlings with CaCl_2_ enhanced damage to membranes, and endogenous H_2_O_2_ concentration under irrigation conditions, but the damage to membranes was more severe and endogenous H_2_O_2_ concentration was greater after the pre-treatment of H_2_O_2_ under similar conditions. However, H_2_O_2_ and CaCl_2_ combined pre-treatment protected the cellular membranes and reduced H_2_O_2_ accumulation upon conditions of drought stress. It could be further interpreted from the data that the endogenous H_2_O_2_ concentration was lowest in seedlings supplemented with 15 mM CaCl_2_. The lower electrolyte leakage and accumulation of endogenous H_2_O_2_ in the seedlings pre-treated by H_2_O_2_ or CaCl_2_ could be due to the activation of ROS scavenging enzymes. Previous studies have indicated an increase in the activities of peroxidase, catalase and the enzymes of water-water cycle after pre-treatment with CaCl_2_. In case of SDS-PAGE procedure, the size of Band 5 was 52 kDa, and the size of Band 12 was found to be almost 12 kDa. Previous studies have suggested that these band sizes correspond to the larger and smaller subunits of rubisco protein respectively [43]. Plants under abiotic stress seem to cause overexpression of this protein, which may play a possible role in plant growth.

## 4. Material and Methods

### 4.1. Plant Material

The greenhouse experiment was carried out at the Institute of Biotechnology and Genetic Engineering (IBGE, Khyber Pakhtunkhwa Agricultural University, and Peshawar in November 2015. Every pot was filled with well-rottedfarm yard manure and silt (1:1). During this experiment, *Brassica napus* ”Bulbul-98” was used. Plants were arranged in a completely randomized design with three replications for precision, and allowed to grow for 35 days after germination. The seedlings were allowed to grow under controlled conditions (light, 100 μmol photon m^–2^·s^–1^; temperature, 25 ± 2 °C; Relative humidity, 65–70%). The plants were sprayed separately two times at two day intervals regularly up to 20 days after germination with the following concentrations of 5, 10 and 15 mM Ca^2+^ in the form of CaCl_2_·2H_2_O) and 2, 5, and 10 μM H_2_O_2_. After 20 days of germination, half of the pots were sufficiently watered and maintained at 100% field capacity (as well-watered), and remaining pots were subjected to drought stress by withholding the water supply at 30% field capacity (as drought stressed). Field capacity was maintained by weighing the pots every day. We included plants with no spray treatment and with irrigation maintained throughout the experiment as extra control treatments.

### 4.2. Rate of Water Loss (RWL)

The rate of water loss was calculated according to the modified Ristic and Jenks (2002) method [44]. Leaf blades were excised from the pots and brought to relative water content (100%) by placing in de-ionized water for 2 h. Excess water was removed, and the leaf blades were weighed by an electric balance. Leaf blades were exposed to air circulation under darkness produced by an electric fan for 500 min. When the leaf blades were measured, the data was taken at different times [Tx (min); x = 0 min]. During exposure to circulating air, leaf blades were weighed at four times and recorded as time Tx where x = 1, 2, 3, and 4. Leaf blades were dried for 48 h at 80 °C and dry mass (DM) was determined. The rate of water loss was calculated as:

Leaf water loss (mg·g^−1^h^−1^ DM) = [(FMTx − FMTx + 1) × 60]/[DM × (Tx + 1 − Tx)]

where FM is fresh mass, DM is dry mass, Tx and Tx + 1 is measuring time.

### 4.3. Relative Water Content

Fresh weight (FW) was obtained by weighing the leaf disc at harvest time. Leaf discs were fully immersed in double distilled water at 4 °C for 24 h. The samples were blotted dry on filter paper after 24 h to determine the turgid weight (TW) by another weighing. Finally, the leaf disc was oven dried at 70 °C for 48 h and dry weight (DW) was obtained. Relative water content was calculated by using the following formula:

Relative Water Content (RWC) = (FW − DW)/(TW − DW) × 100

### 4.4. Chlorophyll Content

The total chlorophyll content was measured according to the method by Arnon [45] by homogenizing leaf samples (100 mg) with 3 mL of 80% acetone. The homogenate were centrifuged at 15,000 rpm and the supernatant was collected. The absorbance were taken at 470, 645 and 663 nm by a UV spectrophotometer (UV 1900, Rayleigh, Beijing Beifen-Ruili Analytical Instrument (Group) Co. Ltd, Beijing, China) according to Lichtenthaler and Wellburn (1983) [46].

### 4.5. Soluble Protein Content

Leaves (100 mg) were homogenized in 2 mL of potassium phosphate buffer (0.05 M, pH 7.4) containing 1 mM PMSF, 2mMdithiothreitol, 0.1 mM (ethylene diamine tetra acetic acid) EDTA, and 20% polyvinyl polypyrrolidone (PVP) using a homogenizer. The sample was then centrifuged at 15,000 rpm. Supernatant was collected and the soluble protein content was quantified using bovine serum albumin as a standard (Bradford 1976) [47].

### 4.6. Electrolyte Leakage

A Consort C-931 conductivity meter was used to measuring the electrolyte leakage in the leaf (5 cm^2^). In 5 mL double distilled water, the leaf discs were incubated at 25 °C for 3 h with shaking and initial conductivity of the solution were determined. After autoclaving the samples, the final conductivity of the solution were determined (100% electrolyte leakage). The quantity of electrolytes leakage were estimated as a percentage (%) of initial to final conductivity.

### 4.7. Proline Content

Proline was measured according to the method by Bates et al. [48]. The plant materials (500 mg) were homogenized in 3% sulphosalicylic acid. The sample was separated by centrifugation at 5000 rpm. At 100 °C for 1 h, 100 μL of the extract was reacted with acid ninhydrin, and then the reaction was terminated in an ice bath. The optical density was measured at 520 nm by mixing the reaction mixture. From a standard curve in the range of 0–20 μg/mL of l-proline, the amount of proline was determined.

### 4.8. H_2_O_2_ Content

Plant materials (100 mg FW) were homogenized with 0.5 mL of trichloroacetic acid (TCA) in an ice bath. The homogenate was centrifuged at 12,000 rpm for 15 min. 100 mM potassium phosphate buffer and 1 M KI were added to each supernatant. Absorbance was measured at 390 nm. H_2_O_2_ was quantified based on a standard curve.

### 4.9. SDS-PAGE

SDS-PAGE gel electrophoresis was done according to the method by Hames et al. [49] by using a 3% stacking and a 15% running gel. Stacking gel: 35.4% (*w*/*v*) acrylamide, 0.62% (*w*/*v*) bis-acrylamide, 10% *w*/*v* SDS, 1M Tris (pH = 6.8), 5 μL of tetra methylene diamine (TEMED), 10% (*w*/*v*) ammonium persulfate (APS) solution, 3.71 mL of distilled water (H_2_O). Running gel: 35.4% (*w*/*v*_ acrylamide, 0.62% (*w*/*v*) bis-acrylamide, 10% *w*/*v* SDS, 1 M Tris (pH = 8.8), 3 mL of distilled water (H_2_O), 6 μL TEMED, and 10% (*w*/*v*) APS solution. The gels were stained with AgNO_3_ solution and rocked at normal room temperature for 30 min. These gels were destained in 10% (*v*/*v*) acetic acid, 3% (*v*/*v*) glycerol, 40% (*v*/*v*) methanol, at normal room temperature.

### 4.10. Statistical Analysis

Analysis of variance (ANOVA) was done by applying the Fisher LSD test with Minitab (17) statistical software. Means with different letters are regarded as statistically significant at *p* ≤ 0.05.

## 5. Conclusions

Drought stress is known to cause disruptions in almost all physiological parameters. However, the exogenous application of various components could help in recovery of the damage caused by this stress. In the current study, we have found that *Brassica* seedlings with drought stress-induced physiological damage recovered with the application of Ca^2+^ and H_2_O_2_ supplementations. Hence, exogenous application of these components could be a suitable strategy to improve crop production under abiotic stress.
